# SERT and uncertainty: serotonin transporter expression influences information processing biases for ambiguous aversive cues in mice

**DOI:** 10.1111/gbb.12215

**Published:** 2015-04-17

**Authors:** S B McHugh, C Barkus, J Lima, L R Glover, T Sharp, D M Bannerman

**Affiliations:** † Department of Experimental Psychology, University of OxfordOxford, UK; ‡ Section on Neuroplasticity, National Institute of Mental Health, National Institutes of HealthBethesda, MD, USA; § Department of Pharmacology, University of OxfordOxford, UK

**Keywords:** 5-HTT, 5-HTTLPR, ambiguous, animal model, cognitive bias, fear, mice, over-expressing, serotonin transporter, SERT

## Abstract

The long allele variant of the serotonin transporter (SERT, 5-HTT) gene-linked polymorphic region (5-HTTLPR) is associated with higher levels of 5-HTT expression and reduced risk of developing affective disorders. However, little is known about the mechanisms underlying this protective effect. One hypothesis is that 5-HTT expression influences aversive information processing, with reduced negative cognitive bias present in those with higher 5-HTT expression. Here we investigated this hypothesis using genetically-modified mice and a novel aversive learning paradigm. Mice with high levels of 5-HTT expression (5-HTT over-expressing, 5-HTTOE mice) and wild-type mice were trained to discriminate between three distinct auditory cues: one cue predicted footshock on all trials (CS+); a second cue predicted the absence of footshock (CS−); and a third cue predicted footshock on 20% of trials (CS20%), and was therefore ambiguous. Wild-type mice exhibited equivalently high levels of fear to the CS+ and CS20% and minimal fear to the CS−. In contrast, 5-HTTOE mice exhibited high levels of fear to the CS+ but minimal fear to the CS− and the CS20%. This selective reduction in fear to ambiguous aversive cues suggests that increased 5-HTT expression reduces negative cognitive bias for stimuli with uncertain outcomes.

The serotonin transporter (SERT or 5-HTT) controls the duration and extent of serotonergic neurotransmission and is the principal target for antidepressant and anxiolytic drugs (Blakely *et al.* 1994; Benarroch 2013). The threefold variation in human 5-HTT expression (Lundberg *et al.* 2007) is thought to be driven, in part, by the 16 or more genetic polymorphisms discovered to date (Hu *et al.* 2006, 2007; Murphy *et al.* 2013), with higher 5-HTT expression associated with long allele homozygotes (LL, specifically the L_A_/L_A_ subtype) compared to carriers of the short allele (short/long, SL or short/short, SS). Moreover, LL homozygotes exhibit reduced neuroticism (Lesch *et al.* 1996), reduced amygdala reactivity to aversive stimuli (Hariri *et al.* 2002, 2005), and a reduced risk of depression (Lesch *et al.* 1996) especially when combined with environmental factors (Caspi *et al.* 2003, 2010; Uher & McGuffin 2010). Notably, LL homozygotes also show reduced negative cognitive bias, with a tendency to avoid aversive stimuli and selectively attend to positive stimuli (Fox *et al.* 2009). This is important because negatively biased information processing is a core feature of affective disorders (Mathews & MacLeod 2005), and it suggests that the reduced negative bias may act as a protective cognitive mechanism (Fox *et al.* 2009; Pergamin-Hight *et al.* 2012).

While human studies have dominated efforts to relate 5-HTT expression to emotional information processing, animal models allow us to investigate neurobiological questions and exert experimental control in a way that is impossible in humans. Here we investigated aversive information processing biases in genetically-modified, 5-HTT over-expressing (5-HTTOE) mice. 5-HTTOE mice have approximately threefold greater SERT expression than their wild-type (WT) counterparts, as verified by radioligand binding of [^3^H]-citalopram (Jennings *et al.* 2006; Barkus *et al.* 2014). This difference mirrors the range of natural 5-HTT variation in humans (Lundberg *et al.* 2007), and is comparable to the proposed difference between the L_A_/L_A_ and SS genotypes (Murphy & Lesch 2008). 5-HTTOE mice therefore provide a more physiologically relevant model of variation in human 5-HTT expression when compared to 5-HTT knock-out rodents, for example, because humans do not exhibit a complete loss of 5-HTT expression.

We devised a task in which WT and 5-HTTOE mice learned to discriminate between three auditory cues: one cue was always paired with shock (CS+), another cue was never paired with shock (CS−) and a third cue was paired with shock on 20% of trials (CS20%). The CS+ and CS− were therefore unambiguous predictors of the presence or absence of shock, respectively, whereas the CS20% predicted both the presence and the absence of shock and therefore remained ambiguous throughout training.

## Materials and methods

### Subjects

This study used 26 male 5-HTTOE mice and 26 male WT littermates bred by mating WT female F1 CBA × C57BL/6 mice (Charles River, Margate, UK) with male 5-HTT OE mice. These mice have been maintained on a CBA × C57BL/6 background since their initial generation, full details of which can be found in Jennings *et al.* (2006). Male mice were used to allow comparisons with our previous investigation of discriminative fear conditioning in 5-HTTOE mice, which was exclusively in males (Barkus *et al.* 2014). Mice were 5–11 months old at the start of testing and housed in a temperature and humidity controlled room under a 12 h light/dark cycle (lights on 0700 h to 1900 h). Testing took place during the light cycle. Mice were housed two to six per cage with *ad libitum* food and water throughout the experiment. The experiments were conducted in accordance with the United Kingdom Animals Scientific Procedures Act (1986) under project licenses PPL 30/2561 and 30/3068 and were approved by local ethical review for the University of Oxford.

### Fear conditioning

Fear conditioning was conducted in one of two operant chambers (ENV-307A, Med Associates Inc., Lafayette, IN, USA), each with distinct visual and olfactory cues. The experiment was carried out over five days which comprised a pre-exposure day, 3 training days and a fear memory recall day (Table 1). Although rare in single-cue fear conditioning experiments, pre-exposure to the auditory cues is commonly performed in discriminative fear conditioning studies (Herry *et al.* 2008; McHugh *et al.* 2013, 2014). Mice were trained to discriminate between three distinct auditory cues that were paired with footshock (0.3 mA, 0.5 seconds) on either 0% (CS−; P(US|CS) = 0), 100% (CS+; P(US|CS) = 1) or 20% of trials (CS20%; P(US|CS) = 0.2). Allocation of the different auditory cues to the CS−, CS+ and CS20% condition was counterbalanced across mice. We chose 20% reinforcement because it is the lowest level of daily partial reinforcement that can be given when using five cue exposures per day (i.e. 1 shock to the CS20% cue per day) and because pilot experiments demonstrated robust discrimination between the CS− and CS20% in WT C57Bl/6 mice.

**Table 1:** Summary of experimental design

Day	Session	Trial types	Total footshocks
1	Pre-exposure	5 × CS−, 5 × CS+, 5 × CS20%	0
2	Training I	5 × CS−→no shock, 5 × CS+→shock, 1 × CS20%→shock, 4 × CS20%→no shock	6
3	Training II	5 × CS−→no shock, 5 × CS+→shock, 1 × CS20%→shock, 4 × CS20%→no shock	6
4	Training III	5 × CS−→no shock, 5 × CS+→shock, 1 × CS20%→shock, 4 × CS20%→no shock	6
5	Fear memory recall	5 × CS−, 5 × CS+, 5 × CS20%	0

Conditioned-stimulus (CS) trial types (CS−, CS+ and CS20%) were pseudo-randomly interleaved on all days. Note that no shocks were given during Pre-exposure (or Fear memory recall) and therefore CS type designations were nominal during pre-exposure.

At the start of each session, two mice (one WT, one 5-HTTOE) were brought to the testing room and each placed into one of two conditioning chambers (context 1 or context 2). One drop of essential oil was placed onto the tissue lining the waste tray to give each context a distinctive smell (almond oil for context 1, lavender for context 2). Following a 300-second lead-in period, mice were presented with 15 auditory cues (5 × 2900 Hz continuous tone, 5 × white noise, 5 × 7000 Hz intermittent tone; all 72 dB and 30 seconds duration) in a pseudo-randomly interleaved order with a mean inter-cue interval of 80 ± 14 seconds (range 60–100 seconds), and with the same cue type never occurring more than twice consecutively within a session. During training, the CS+ was always followed by footshock (5/5 shock trials per day; 15 shock trials in total); the CS− was never followed by footshock (zero shock trials in total); and the CS20% was followed by footshock on 20% of trials (1/5 shock trials per day; three shock trials in total). The position of the shocked CS20% trial varied from day-to-day so that the mice did not condition to trial order. No shocks were administered at all during the pre-exposure or fear memory recall sessions. At the end of each session, mice were removed from the chamber, which was then cleaned with 10% ethanol alcohol, and a fresh tissue placed into the waste tray. The total session length was 1930 seconds (32.5 min).

Note that for a given mouse the pre-exposure and training sessions were performed in one chamber (e.g. context 1, counterbalanced across mice) and the fear memory recall session was performed in the other (novel) chamber (e.g. context 2 if trained in context 1). During fear conditioning, mice condition not only to the discrete auditory cues but also to the training context. Testing fear memory recall in a novel chamber therefore provides a test of cue-evoked fear unconfounded by contextual fear conditioning.

### Data analyses

Freezing behavior was measured using a script in NIH Image (Schneider *et al.* 2012), which compared consecutive video frames (1 Hz sampling) for pixel changes and assigned a freezing score if the % pixel change was below a set threshold calibrated for an absence of movement except for breathing. This automated system has over 80% correlation with human ratings of freezing behavior and gives a completely unbiased measure of immobility. A detailed description can be found in Richmond *et al.* (1998).

To analyze cue-evoked freezing responses, we calculated the percentage freezing in the 30 seconds before cue-onset and subtracted this from the percentage freezing during cue presentation (i.e. % CS freezing minus % pre-CS freezing). Therefore every cue presentation had its own baseline and cue-evoked freezing is presented as a difference score. For example, if a mouse froze for 22 seconds during the 30 seconds cue (22/30 = 73.3%) and 11 seconds during the 30 seconds pre-CS period (11/30 = 36.7%) this would constitute a ‘freezing difference score’ of 73.3 − 36.7 = 36%. Positive difference scores indicate increased freezing compared to the pre-stimulus period whereas negative difference scores indicate decreased freezing compared to the pre-stimulus period. Note that analysis of cue-evoked freezing responses on the first training day excluded responses to the first CS+, CS− and CS20% trial because they could have occurred before the first shock had been given. Additional analyses of raw pre-CS freezing and raw CS-evoked freezing can be found in the supplementary material.

To analyze freezing responses due to contextual conditioning, we calculated the percentage freezing during the 300 seconds lead-in period that occurred at the start of each session. We examined three specific time points: (1) before fear conditioning (i.e. the 300 seconds lead-in period during the pre-exposure session); (2) after at least one fear conditioning session (i.e. the 300 seconds lead-in period averaged over training days 2 and 3); (3) after fear conditioning but in a novel context not associated with shock (i.e. the 300 seconds lead-in period during the fear memory recall session).

### Statistical analysis

Data were analyzed using analysis of variance (anova) in spss (version 22, IBM, Armonk, NY, USA). Analyses of variance are described using a modified version of Keppel's (1982) notation in which the dependent variable is defined in the form: A_2_ × B_3_ × S_51_, where A is a factor with two levels, B a factor with three levels, and S_51_ denotes that 51 subjects were included in the analysis. One WT mouse was subsequently removed from analysis due to an indeterminate genotyping result post-experiment. Final numbers for analyses were 25 WT and 26 5-HTTOE mice.

## Results

### 5-HTTOE mice show reduced freezing to an ambiguous cue during fear learning

**Figure 1:**
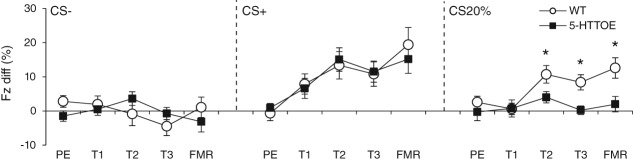
Conditioned-stimulus (CS)-evoked freezing responses in wild-type (WT; white circles) and serotonin transporter over-expressing mice (5-HTTOE; black squares) during pre-exposure (PE), training days 1–3 (T1, T2, T3) and fear memory recall (FMR) CS− (left panel) and CS+ (middle panel) evoked responses did not differ between genotypes whereas CS20% (right panel) evoked responses differed from T2 onwards. Freezing is plotted as a difference score (freezing during the 30 seconds of cue presentation minus freezing in the 30 seconds before cue presentation), with negative scores indicating less freezing during the cue compared to the 30 seconds before the cue. *P < 0.01.

Neither WT nor 5-HTTOE mice froze during presentation of any of the cues in the pre-exposure session (Fig. 1; anova model: genotype_2_ × CS type_3_ × trial_5_ × S_51_; all *F* < 1.4, all *P* > 0.2). On the first training day, although both WT and 5-HTTOE mice froze more during CS+ than either CS− or CS20% trials, there were no differences between the genotypes (see Figs. S1–S3 and Supporting Information for statistical analyses). However, during training days 2 and 3, a striking difference between the genotypes emerged. Whereas WT mice exhibited high levels of cue-evoked freezing to both the CS+ and the CS20% compared to the CS−, 5-HTTOE mice exhibited high levels of cue-evoked freezing only to the CS+, with low levels of freezing to both the CS20% and the CS− (Fig. 1).

Statistical analyses confirmed these observations (anova model: genotype_2_ × day_2_ × CS type_3_ × trial_5_ × S_51_), with an interaction between genotype and CS type (*F*_2,98_ = 3.1, *P* = 0.048). WT mice froze significantly more during CS+ and CS20% trials compared to CS− trials (both *P* < 0.001), with no difference between CS+ and CS20% trials (*P* = 0.8). 5-HTTOE mice froze more during CS+ trials than either CS− or CS20% trials (both *P* = 0.003), with no difference between CS− and CS20% trials (*P* = 0.5). Importantly, WT mice froze significantly more than 5-HTTOE mice during CS20% trials (*P* = 0.002), whereas freezing levels did not differ between the genotypes during CS− (*P* = 0.2) or CS+ trials (*P* = 0.8). Thus higher SERT expression resulted in selectively reduced freezing to the ambiguous CS20% cue.

### 5-HTTOE mice show reduced freezing to an ambiguous cue during fear memory recall

A similar pattern of freezing responses was seen during fear memory recall, during a session in a novel context in which the auditory cues were presented but no shocks were given (Fig. 1). Both genotypes exhibited high levels of cue-evoked freezing during CS+ trials and low levels of freezing during CS− trials, but 5-HTTOE mice froze significantly less than WTs during CS20% trials. To mitigate the effects of extinction, we restricted analysis to the first two trials of each CS type during the fear memory recall session. Analyses of variance (model: genotype_2_ × CS type_3_ × trial_2_ × S_51_) revealed main effects of CS type (*F*_2,98_ = 11.6, *P* < 0.001) and genotype (*F*_1,49_ = 7.2, *P* = 0.01), with lower freezing in 5-HTTOE mice.

Although the interaction term was not significant (*F*_2,98_ = 0.5, *P* = 0.6), planned comparisons were justified based on the *a priori* prediction given by the results on training days 2 and 3. These comparisons revealed that WT mice froze more during CS+ and CS20% trials compared to CS− trials (*P* = 0.004 and *P* = 0.009, respectively), whereas 5-HTTOE mice froze more during CS+ trials compared to both CS− and CS20% trials (*P* = 0.003 and *P* = 0.03, respectively). Importantly, WT mice froze significantly more than 5-HTTOE mice during CS20% trials (*P* = 0.006), whereas responses to the CS− and CS+ did not differ between genotypes (*P* = 0.3 and *P* = 0.5, respectively). In short, the selective reduction in cue-evoked freezing during the ambiguous CS20% cue was present during fear memory recall as well as during training.

### 5-HTTOE mice show reduced freezing to the conditioning context

**Figure 2:**
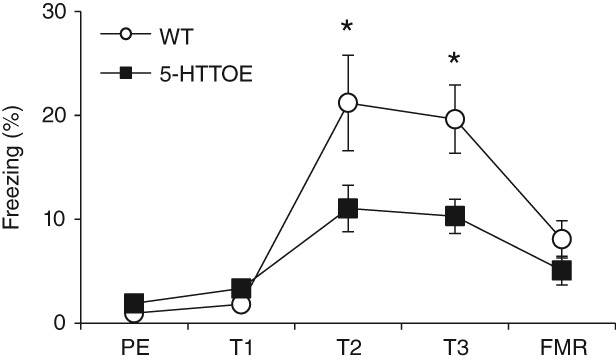
Context-evoked freezing responses in WT (white circles) and 5-HTTOE mice (black squares) in the 300 seconds before the auditory cues were presented during the pre-exposure (PE), training days 1–3 (T1, T2, T3) and fear memory recall (FMR) sessions *P < 0.05.

At the start of each session there was a 300-second lead-in period before any auditory cues were presented. Analysis of freezing during this period allowed us to investigate context-driven fear responses at three distinct phases of the experiment: (1) before conditioning took place (i.e. the pre-exposure session); (2) after fear conditioning (i.e. on training days 2 and 3), and (3) after fear conditioning but in a novel context (i.e. the fear memory recall session). During the pre-exposure session, freezing levels were low in both genotypes, and similar in WT and 5-HTTOE mice (Fig. 2). On training days 2 and 3, freezing levels were much higher in both genotypes, and were significantly higher in WT than 5-HTTOE mice. During the fear memory recall session, freezing levels were lower in both genotypes, and again similar in WT and 5-HTTOE mice (Fig. 2).

Statistical analyses confirmed these observations. Analyses of variance (model: genotype_2_ × training phase_3_ × S_51_) revealed a genotype × training phase interaction (*F*_2,98_ = 4.2, *P* = 0.02). Simple main effects analysis revealed significant effects of training phase in both WT (*F*_2,48_ = 25.7; *P* < 0.001) and 5-HTTOE mice (*F*_2,48_ = 5.4; *P* = 0.008), with higher freezing in both groups during training compared to pre-exposure (WT: *P* < 0.001; 5-HTTOE: *P* = 0.004). Importantly, pairwise comparisons revealed higher freezing in WT than 5-HTTOE mice on training days 2 and 3 (*P* = 0.02) but not during pre-exposure (*P* = 0.1) nor in the novel context used for the fear memory recall session (*P* = 0.2). Thus, 5-HTTOE mice also exhibited reduced contextual conditioning.

## Discussion

### Summary of results

Compared to WTs, 5-HTTOE mice exhibited decreased fear-related behavior (freezing) to an ambiguous cue that predicted an aversive outcome on 20% of trials (CS20% cue). 5-HTTOE mice also exhibited decreased freezing to the conditioning context, consistent with an earlier report (Line *et al.* 2014). This reduced fear-related behavior did not reflect an inability to express appropriate cue-evoked freezing responses because WT and 5-HTTOE mice exhibited equivalently high levels of freezing to the CS+, which reliably predicted footshock, and equivalently low levels of freezing to the CS−, which reliably predicted the absence of footshock. Like the CS20% cue, the training context can also become associated with both the presence and the absence of footshock, making it an ambiguous predictor. The reduced freezing to the CS20% cue and the training context may therefore reflect a common mechanism. Our results suggest that 5-HTT expression strongly influences how ambiguous aversive cues are processed.

### Reduced negative bias or reduced learning rate?

Our interpretation of the present data is that 5-HTTOE mice exhibit reduced negative bias because they treat the CS20% in a similar way to the CS− whereas WTs treat the CS20% in a similar way to the CS+. Alternatively, it could be that the rate of learning is simply slower in 5-HTTOE mice. We cannot rule out this explanation and, indeed, the two accounts may not be incompatible. For example, reduced negative bias may reflect a reduced tendency to form associations between stimuli and aversive outcomes, especially when they are only occasionally paired together. Nevertheless, it is important to note that 5-HTTOE mice exhibit normal learning rates and normal memory retrieval in the water maze, a classical test of spatial reference memory, and on the appetitive elevated Y-maze reference memory task (Line *et al.* 2014), suggesting that 5-HTTOE mice do not generally exhibit slower learning.

Moreover, our data cannot be explained by hearing impairments, altered response to the footshock, or changes in locomotor activity in 5-HTTOE mice. Acoustic startle responses and the unconditioned response to the auditory cues and the footshock are normal in 5-HTTOE mice (Barkus *et al.* 2014). Furthermore, locomotor activity is slightly reduced in 5-HTTOE mice compared to WTs, which might predict increased rather than decreased freezing (Line *et al.* 2011). Thus the decreased freezing to ambiguous cues is unlikely to be due to some general sensory or motor consequence of 5-HTT over-expression.

### 5-HTT over-expressing mice and anxiety

The deficit for processing ambiguous aversive cues in the present study is consistent with the reduced anxiety shown by 5-HTTOE mice in unconditioned tests such as the elevated plus maze and food neophobia (Jennings *et al.* 2006; Line *et al.* 2011). These tasks generate anxiety by placing the mouse into situations of uncertainty, where there is conflict between competing goals. For example, the elevated plus maze exploits the approach/avoidance conflict in mice between their natural preference for dark, enclosed spaces vs. their instinctive drive to explore novel environments. WT mice spend more time in the enclosed (safe) arms of the maze and are slower to enter the exposed (more anxiogenic) arms compared to 5-HTTOE mice. The uncertainty of the situation (i.e. not knowing whether the environmental cues predict safety or danger) may be a necessary condition to generate anxiety in tasks like the elevated plus maze (File *et al.* 1993). Therefore, reduced negative bias in processing these ambiguous cues may also underlie the reduced anxiety in 5-HTTOE mice.

### Neurobiology of 5-HTT over-expressing mice

So what are the neurobiological features of 5-HTTOE mice that could underpin their behavioral phenotype? The amygdala plays a key role in processing aversive cues (Davis 1992; Davis & Whalen 2001), and previously we have shown that amygdala function is altered in 5-HTTOE mice (Barkus *et al.* 2014). Using a discriminative fear conditioning paradigm with a CS+ and CS− (but no CS20% cue), we found that amygdala hemodynamic responses evoked by the CS+ were significantly lower in 5-HTTOE compared to WT mice. These findings are consistent with human 5-HTTLPR imaging studies, which report reduced amygdala hemodynamic responses to aversive stimuli in the high SERT expressing LL genotype (Hariri *et al.* 2002, 2005). Fear-evoked amygdala theta power was also reduced in 5-HTTOE mice (Barkus *et al.* 2014). Theta activity is rhythmical neuronal activity between 5 and 10 Hz, hypothesized to facilitate synaptic plasticity (Sejnowski & Paulsen 2006). So amygdala function is altered in 5-HTTOE mice.

However, some aspects of the 5-HTTOE phenotype are also consistent with altered hippocampal function. For example, reduced freezing responses to partially but not fully predictive aversive cues have been reported in a mouse model with impaired hippocampal function (Tsetsenis *et al.* 2007). Also, reduced anxiety on tasks like the elevated plus maze and food neophobia is commonly seen in rodents with ventral hippocampal lesions (Bannerman *et al.* 2003, 2004; McHugh *et al.* 2004). One possibility is that communication between the amygdala and ventral hippocampus may gate fear/anxiety responses to ambiguous conditioned stimuli, as recently reported during anxiety tasks (Felix-Ortiz *et al.* 2013).

### Serotonin and aversive prediction errors

Theoretical models have long posited that serotonergic neurons may encode aversive prediction errors, which are believed to be a necessary condition for fear learning (Daw *et al.* 2002; Dayan & Huys 2009). Moreover, recent data have shown that rats with chemical lesions of 5-HT neurons in the dorsal raphe nucleus (DRN) display greater fear-related behavior (conditioned suppression) than controls to an ambiguous cue, with no differences in conditioned suppression to a fully predictive cue (CS+) or a cue that never predicts shock (CS−) during discrimination learning (Berg *et al.* 2014). The authors suggest that DRN-lesions impair the ability to use negative prediction errors (NPEs), which occur on trials when the ambiguous cue is not followed by shock. They argue that CS− evoked responses, which are not affected by DRN-lesions, do not require NPEs because the rats can distinguish the CS− from the CS+ based on the distinct sensory properties of the cues.

Prima facie, the deficit in 5-HTTOE mice and DRN-lesioned rats is similar in that both respond appropriately to the CS+ and CS− cues but not to ambiguous, partially reinforced cues. However, whereas DRN-rats tend to over-respond to ambiguous cues (i.e. greater conditioned suppression), 5-HTTOE mice showed a decreased response (i.e. reduced freezing). DRN-lesions and 5-HTTOE are different manipulations, although both appear to reduce 5-HT signaling: DRN-lesions destroy 5-HT neurons and cause a loss of 5-HT tone in DRN-projecting brain regions, whereas 5-HTTOE is associated with reduced tissue levels of 5-HT and reduced extracellular levels of evoked 5-HT (Jennings *et al.* 2010). It is unclear why DRN-lesions and 5-HTTOE produce opposite effects in processing ambiguous aversive cues. One possibility is that DRN-lesions bias 5-HT signaling (and hence control of behavior) to median raphe-targeted structures such as the hippocampus, although this remains to be tested. However, it could also reflect developmental aspects. For example, the behavioral phenotype of adult 5-HTT knock-out mice is partly driven by altered 5-HT function during a critical period of development rather than ongoing changes in 5-HT in the adult (Ansorge *et al.* 2004).

### Cognitive bias in mice and humans

In humans, anxiety and depression are both associated with increased negative cognitive biases and prospective studies show that these are not simply a consequence of affective illness (Mathews & MacLeod 2005). Cognitive bias studies of the 5-HTTLPR have most commonly used the dot-probe task, in which two stimuli (e.g. an aversive picture vs. a neutral or positive picture) are simultaneously presented on a screen and, after they disappear, a dot appears in the same position as one of the stimuli. The participant has to press a button as fast as possible when the dot appears. Negative cognitive bias is reflected by faster reaction times when the dot appears in the same position as the aversive picture (compared to the neutral or positive picture). The LL genotype is associated with reduced negative bias compared to the SS and SL genotypes (Pergamin-Hight *et al.* 2012), consistent with the present study. Although our fear conditioning paradigm and the dot-probe task are clearly very different, both tasks seek to measure responses to a potentially aversive (essentially ambiguous) cue. Although other cognitive bias tasks have been developed for rodents, they are more complex and require extensive training compared to our paradigm (Enkel *et al.* 2010; Brydges *et al.* 2011; Anderson *et al.* 2013). Our task is simple and rapidly acquired and the CS+ and CS− cues act as important within-subject controls. Moreover, with some modification it could also be used in humans.

## Conclusion

Reduced negative bias may act as a protective mechanism for affective disorders and is influenced by 5-HTTLPR genotype. This study demonstrates that mice with increased 5-HTT expression exhibit reduced negative bias for ambiguous aversive cues and represents an important first step for subsequent investigations into the neuronal mechanisms by which 5-HTT expression influences cognitive bias.

## Supporting Information

**Figure S1:** Raw freezing responses in WT and 5-HTTOE mice showing pre-CS freezing (white bars for WT, light gray for 5-HTTOE) and during-CS freezing (black bars for WT, dark gray for 5-HTTOE) for the three cue types (CS−, CS+, CS20%). (a) Responses during pre-exposure. (b) Responses during training day 1. (c) Responses averaged over training days 2 and 3. (d) Responses during the first two trials of the fear memory recall session. **P* < 0.05; n.s.: non-significant.

**Figure S2:** Raw pre-CS freezing responses inWT and 5-HTTOE mice across all trials of the experiment for CS− (upper panel), CS+ (middle panel), and CS20% (lower panel).

**Figure S3:** Raw cue-evoked freezing responses inWT and 5-HTTOE mice across all trials of the experiment for CS− (upper panel), CS+ (middle panel), and CS20% (lower panel).

**Appendix S1:** Supplementary results.
