# Supplementary material for: SERT and uncertainty: serotonin transporter expression influences information processing biases for ambiguous aversive cues in mice
Source: Genes Brain Behav. 2015 Apr 17;14(4):330–6. doi: 10.1111/gbb.12215 (PMC4440341; doi:10.1111/gbb.12215)
Supplement: Supplementary file 1 — Figure S1: Raw freezing responses in WT and 5-HTTOE mice showing pre-CS freezing (white bars for WT, light gray for 5-HTTOE) and during-CS freezing (black bars for WT, dark gray for 5-HTTOE) for the three cue types (CS−, CS+, CS20%). (a) Responses during pre-exposure. (b) Responses during training day 1. (c) Responses averaged over training days 2 and 3. (d) Responses during the first two trials of the fear memory recall session. *P < 0.05; n.s.: non-significant. [file gbb0014-0330-sd1.pdf]

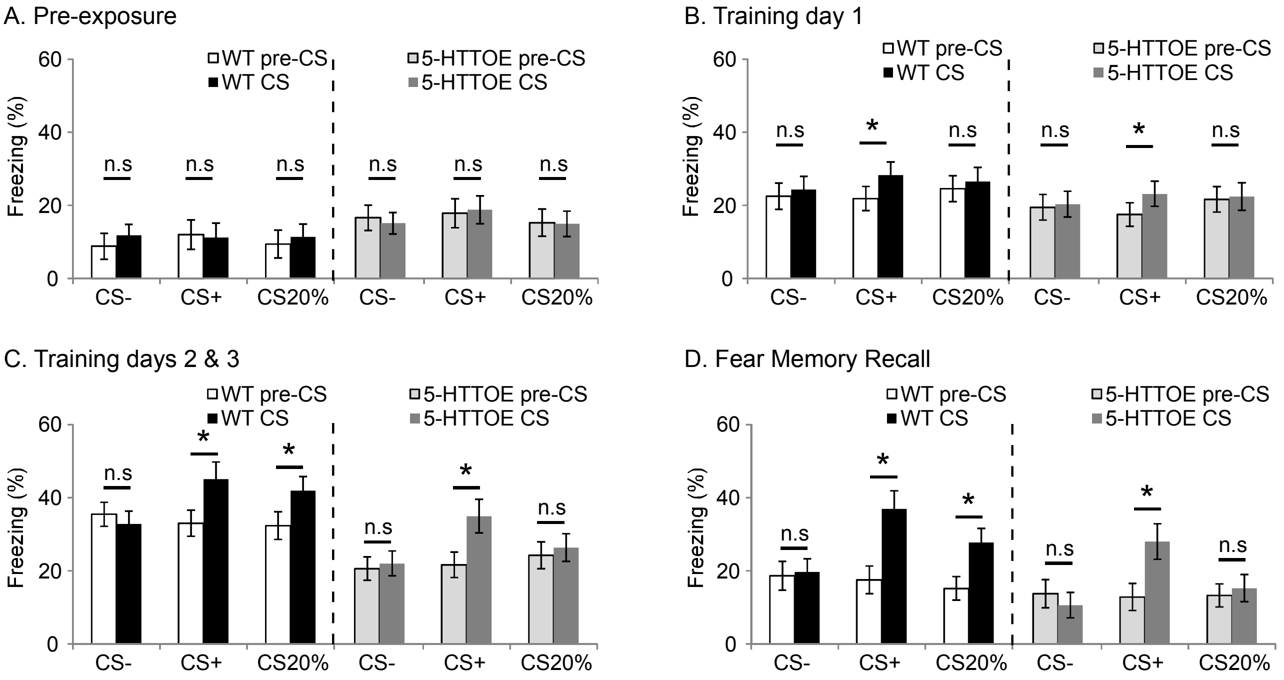

Supplementary Figure S1. Raw freezing responses in WT and 5-HTTOE mice showing pre-CS freezing (white bars for WT, light gray for 5-HTTOE) and during-CS freezing (black bars for WT, dark gray for 5-HTTOE) for the three cue types (CS-, CS+, CS20%). A. Responses during pre-exposure. B. Responses during training day 1. C. Responses averaged over training days 2 and 3. D. Responses during the first two trials of the fear memory recall session. *$p < 0.05$; n.s: non-significant.
