# Supplementary material for: SERT and uncertainty: serotonin transporter expression influences information processing biases for ambiguous aversive cues in mice
Source: Genes Brain Behav. 2015 Apr 17;14(4):330–6. doi: 10.1111/gbb.12215 (PMC4440341; doi:10.1111/gbb.12215)
Supplement: Supplementary file 2 — Figure S2: Raw pre-CS freezing responses inWT and 5-HTTOE mice across all trials of the experiment for CS− (upper panel), CS+ (middle panel), and CS20% (lower panel). [file gbb0014-0330-sd2.pdf]

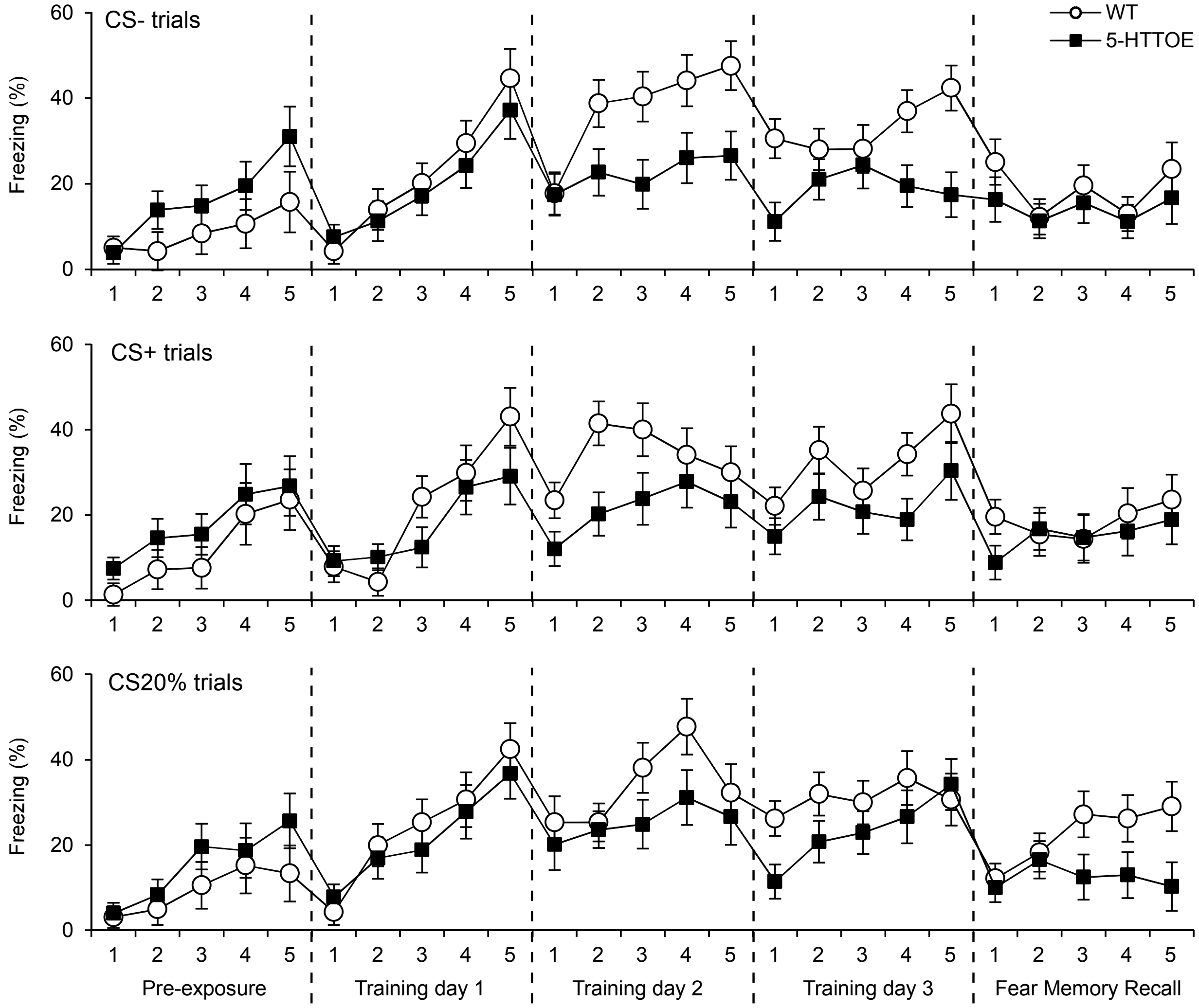

Supplementary Figure S2. Raw pre-CS freezing responses in WT and 5-HTTOE mice across all trials of the experiment for CS- (upper panel), CS+ (middle panel), and CS20% (lower panel).
