# Supplementary material for: SERT and uncertainty: serotonin transporter expression influences information processing biases for ambiguous aversive cues in mice
Source: Genes Brain Behav. 2015 Apr 17;14(4):330–6. doi: 10.1111/gbb.12215 (PMC4440341; doi:10.1111/gbb.12215)
Supplement: Supplementary file 4 — Appendix S1: Supplementary results. [file gbb0014-0330-sd4.pdf]

Supplementary Material

SERT and uncertainty: serotonin transporter expression influences information processing biases for ambiguous aversive cues in mice

Stephen B. McHugh, Christopher Barkus, Joao Lima, Lucas R. Glover, Trevor Sharp, David M. Bannerman

Contents:

Supplementary Figure S1 2

Supplementary Results 3

Supplementary Figure S2 5

Supplementary Figure S3 6

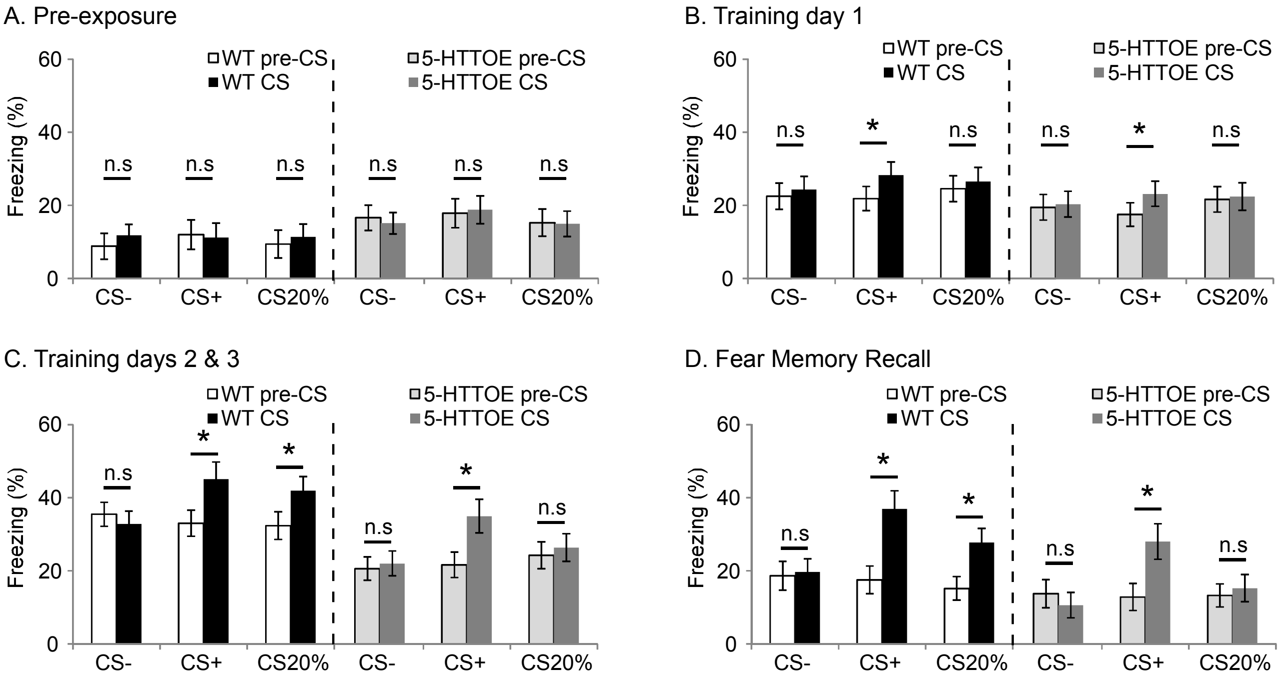

Supplementary Figure S1. Raw freezing responses in WT and 5-HTTOE mice showing pre-CS freezing (white bars for WT, light gray for 5-HTTOE) and during-CS freezing (black bars for WT, dark gray for 5-HTTOE) for the three cue types (CS-, CS+, CS20%). A. Responses during pre-exposure. B. Responses during training day 1. C. Responses averaged over training days 2 and 3. D. Responses during the first two trials of the fear memory recall session. *$p < 0.05$; n.s: non-significant.

Supplementary Results

Figure S1 shows raw pre-cue (pre-CS) and cue-evoked (CS) freezing responses for all days of the experiment. We first analysed these freezing responses for the pre-exposure day (ANOVA model: $genotype_2 \times phase_{(pre\text{-}CS,during\ CS)\ 2} \times CS\ type_3 \times trial_5 \times S_{51}$). There was a main effect of trial ($F(4,196) = 10.6$, $P < 0.001$), reflecting the increase in immobility over the course of the session, but there were no other main effects or interactions (see Figure S1A).

Performing the same analysis on training day 1 revealed main effects of phase ($F(1,49) = 10.6$, $P = 0.002$) and trial ($F(4,196) = 48.2$, $P < 0.001$), and an interaction between phase and CS type ($F(2,98) = 3.8$, $P = 0.025$). Simple main effects analysis of the effect of phase within each CS type revealed higher freezing during CS+ trials compared to the pre-CS+ period ($F(1,49) = 12.1$; $P = 0.001$), and this effect was equivalent in both genotypes, whereas there was no effect of phase for the CS- or CS20% trials ($F(1,49) < 1.2$; $P > 0.25$). There were no other main effects or interactions. These data are summarized in Figure S1B.

An analysis spanning training days 2 and 3 (ANOVA model: $genotype_2 \times phase_{(preCS,during\ CS)\ 2} \times day_2 \times CS\ type_3 \times trial_5 \times S_{51}$) revealed a three-way interaction between genotype, phase, and CS type ($F(2,98) = 3.1$, $P = 0.048$). Simple main effects analysis in the 5-HTTOE mice revealed a main effect of phase in CS+ trials ($F(1,49) = 16.9$, $P < 0.001$) but not in CS- or CS20% trials (F (1,49) < 1.8, $P = 0.2$). In contrast, in WT mice there was a main effect of phase in both the CS+ ($F(1,49) = 13.4$, $P = 0.001$) and CS20% trials ($F(1,49) = 35.5$, $P < 0.001$) but not in the CS- trials ($F(1,49) = 1.4$, $P = 0.2$). These data are summarized in Figure S1C.

Finally, we analysed pre-CS and CS freezing levels for the first two trials of each type during the fear memory recall day (ANOVA model: $genotype_2 \times phase_{(preCS,during\ CS)\ 2} \times CS\ type_3 \times$

trial$_2$ × S_{51}). This analysis revealed main effects of phase ($F(1,49) = 44.2$, $P < 0.001$) and CS type ($F(2,98) = 6.1$, $P = 0.003$), as well as interactions between CS type and phase ($F(2,98) = 11.6$, $P < 0.001$) and genotype and phase ($F(1,49) = 7.2$, $P = 0.01$). Although the three-way interaction between genotype, CS type, and phase was not significant ($F(2,98) = 0.5$, $P = 0.6$), simple main effects analysis were informative about how 5-HTTOE and WT mice changed their freezing responses in the presence of the cues. 5-HTTOE mice increased their freezing to the CS+ cue (effect of phase for CS+: $F(1,49) = 11.0$, $P = 0.002$) but not the CS20% cue (effect of phase for CS20%: $F(1,49) = 0.6$, $P = 0.5$). In contrast, WT mice increased their freezing to both the CS+ cue (effect of phase for CS+: $F(1,49) = 17.3$, $P < 0.001$) and the CS20% cue (effect of phase for CS20%: $F(1,49) = 22.4$, $P < 0.001$). These data are summarized in Figure S1D.

Raw freezing responses from the pre-CS and during-CS periods for all trials are shown in Figure S2 and S3, respectively. Note that raw freezing responses were lower in 5-HTTOE mice versus WTs during both pre-CS and CS periods during training days 2 and 3. The key point is that the change in freezing from pre-CS to during-CS was equivalent in WT and 5-HTTOE mice for CS- and CS+ trials but was significantly greater in WT than 5-HTTOE mice for CS20% trials, as shown in Figure S1C.

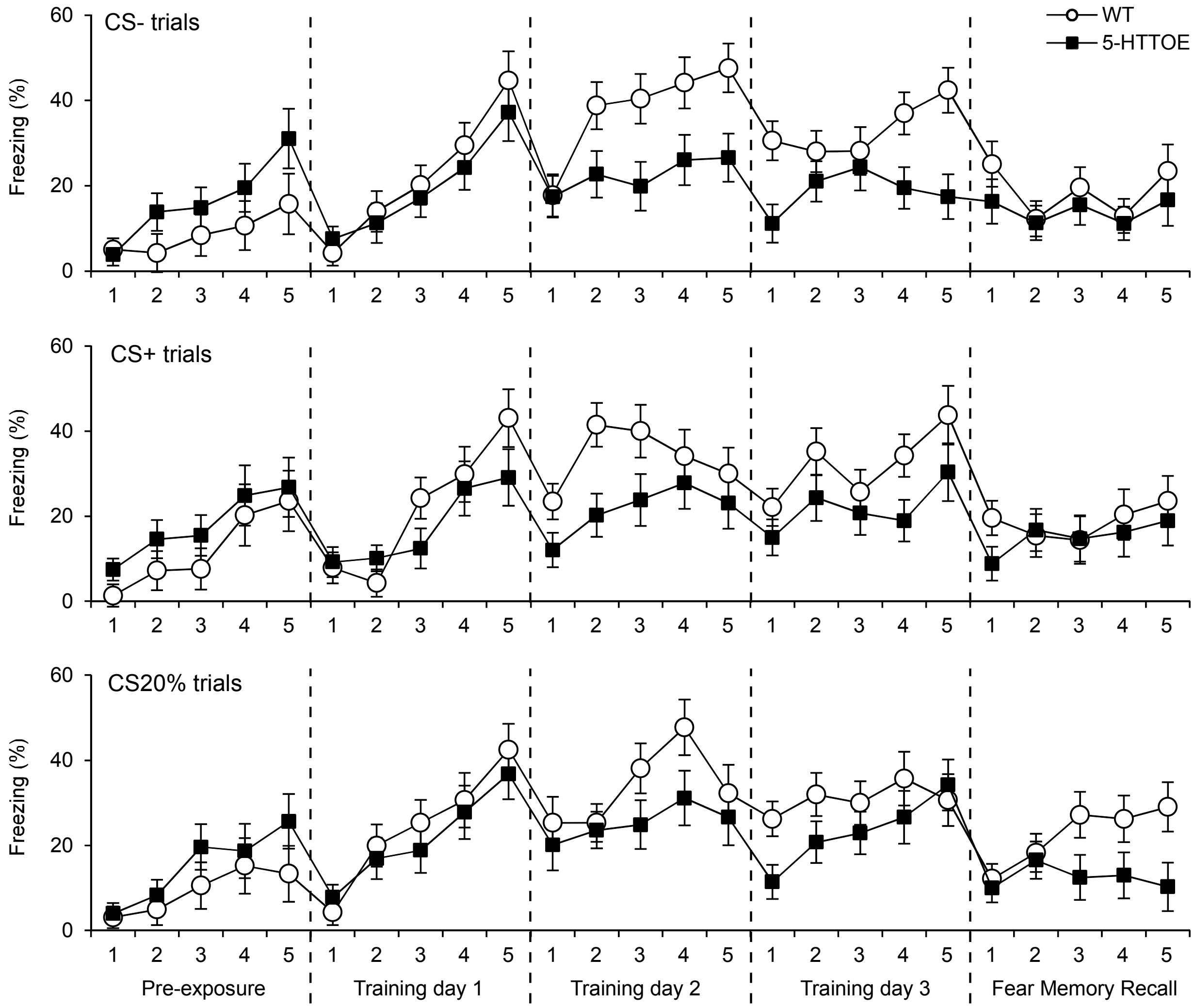

Supplementary Figure S2. Raw pre-CS freezing responses in WT and 5-HTTOE mice across all trials of the experiment for CS- (upper panel), CS+ (middle panel), and CS20% (lower panel).

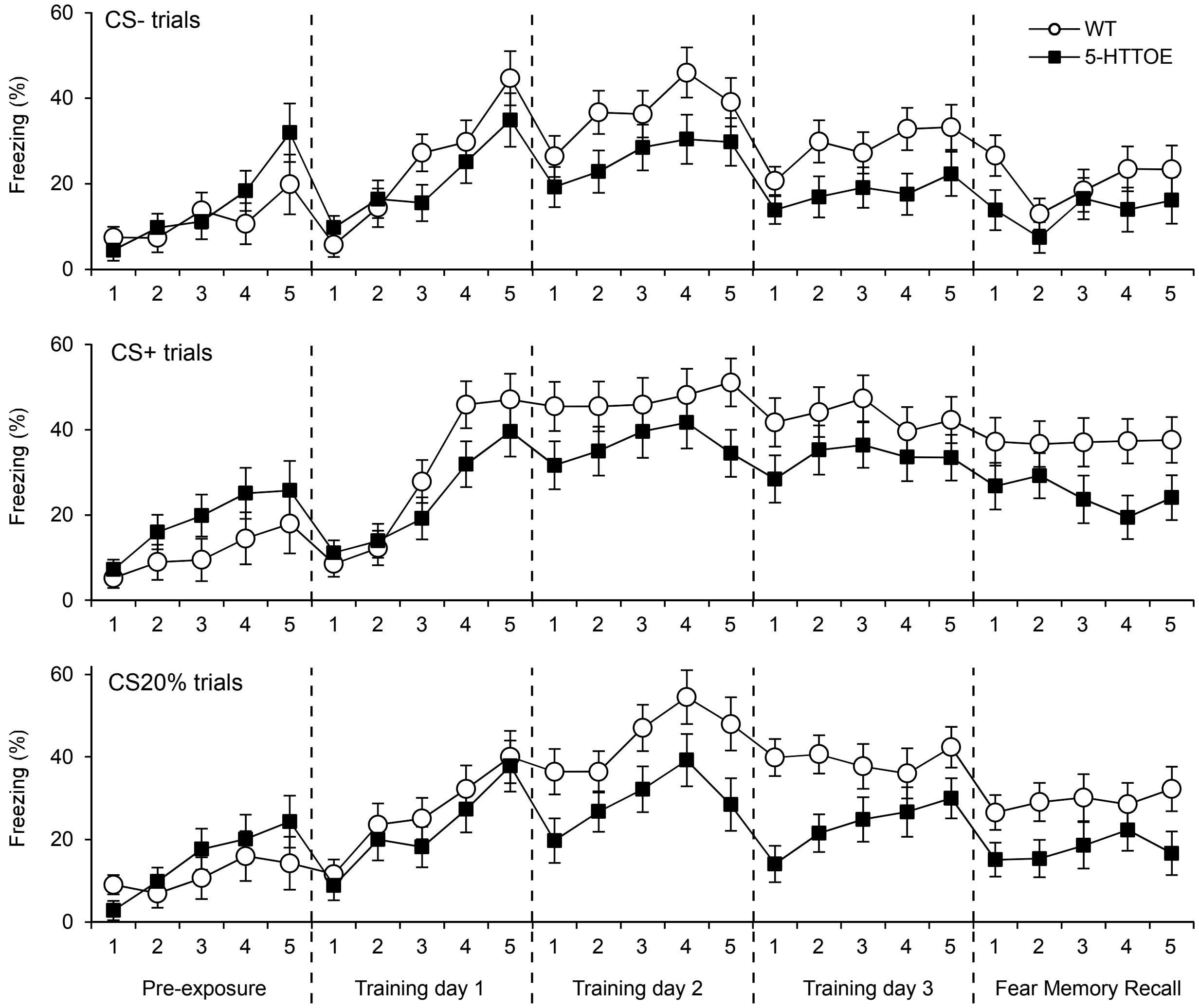

Supplementary Figure S3. Raw cue-evoked freezing responses in WT and 5-HTTOE mice across all trials of the experiment for CS- (upper panel), CS+ (middle panel), and CS20% (lower panel).
